# Inhibition of TRPC3-Nox2 Complex Formation Ameliorates Skeletal Muscle Atrophy

**DOI:** 10.3390/antiox15010038

**Published:** 2025-12-26

**Authors:** Yuri Kato, Di Wu, Tomoya Ito, Yara Atef, Koichi Ayukawa, Xinya Mi, Kazuhiro Nishiyama, Akiyuki Nishimura, Motohiro Nishida

**Affiliations:** 1 Graduate School of Pharmaceutical Sciences, Kyushu University, Fukuoka 812-8582, Japan; yu-kato@phar.kyushu-u.ac.jp (Y.K.); wu.di.536@s.kyushu-u.ac.jp (D.W.); t.ito@phar.kyushu-u.ac.jp (T.I.); ahmed.mohamed.atef.ahmed.sayed.yara.849@m.kyushu-u.ac.jp (Y.A.); mixinya@phar.kyushu-u.ac.jp (X.M.); 2 Laboratory of Prophylactic Pharmacology, Graduate School of Veterinary Science, Osaka Metropolitan University, Osaka 598-8531, Japan; knishiyama@omu.ac.jp; 3 Division of Cardiocirculatory Signaling, National Institute for Physiological Sciences & Exploratory Research Center on Life and Living Systems, National Institutes of Natural Sciences, Okazaki 444-8787, Japan; aki@nips.ac.jp; 4 Exploratory Research Center on Life and Living Systems (ExCELLS), National Institutes of Natural Sciences, Okazaki 444-8787, Japan; 5 Department of Physiological Sciences, SOKENDAI (School of Life Science, The Graduate University for Advanced Studies), Okazaki 444-8787, Japan

**Keywords:** skeletal muscle atrophy, TRPC3, Nox2, protein–protein interaction

## Abstract

Skeletal muscle atrophy underlies sarcopenia, frailty, and muscular dystrophies, but the molecular mechanisms linking oxidative stress to muscle degeneration remain incompletely understood. We previously identified protein complex formation between transient receptor potential canonical 3 (TRPC3) and NADPH oxidase 2 (Nox2) as a key driver of anthracycline-induced myocardial atrophy. Here, we investigated whether this complex also contributes to skeletal muscle wasting. In skeletal muscle from sciatic nerve transection model mice and Duchenne muscular dystrophy (*mdx*) mice, TRPC3-Nox2 complex formation was enhanced. TRPC3 deletion significantly attenuated denervation-induced soleus atrophy and reduced reactive oxygen species (ROS) production. TRPC3-Nox2 complex formation was upregulated in the soleus muscle (SM) of *mdx* mice. Pharmacological disruption of the TRPC3-Nox2 interaction improved muscle size and strength and reduced plasma creatine kinase in *mdx* mice. A recombinant adeno-associated virus (AAV) encoding a TRPC3 C-terminal peptide was used to suppress TRPC3-Nox2 complex formation in vivo. AAV-mediated expression of TRPC3 C-terminal peptide mitigated muscle wasting (CSA) in *mdx* mice, while muscle strength and plasma CK were not significantly improved. Thus, TRPC3-Nox2 complex formation may be a pivotal driver of oxidative stress-mediated skeletal muscle atrophy. Targeting this protein–protein interaction represents a promising therapeutic strategy for Duchenne muscular dystrophy (DMD) and other intractable muscle-wasting disorders.

## 1. Introduction

Striated muscles, including cardiac and skeletal muscles, are essential for movement, blood circulation, and energy metabolism [1]. Muscle atrophy associated with aging and disease contributes to sarcopenia and frailty [2,3]. Muscular dystrophies are genetic disorders characterized by progressive muscle weakness and wasting [4]. In the United States, approximately 45% of older adults (aged 60 years or older) are reported to exhibit sarcopenia [5]. Even a 10% reduction in its prevalence could save over USD 1.1 billion annually in healthcare costs [6]. The prevalence of muscular dystrophy has been reported to range from 19.8 to 25.1 per 100,000 person-years [7]. Therefore, the economic impact of muscle wasting is significant and has become a critical issue directly linked to patients’ QOL. Recent treatment approaches for muscle atrophy include exercise, nutritional therapy, antisense oligonucleotides, and AAV gene therapy approaches, but there is still no fundamental treatment, and there is a need to develop new therapeutic targets and drugs [4].

Disrupted Ca^2+^ handling in skeletal muscle reportedly contributes to muscle dystrophy [8,9,10]. The increase in intracellular Ca^2+^ concentration is caused by rapid release from sarcoplasmic reticulum (SR) and Ca^2+^ influx across the plasma membrane through Ca^2+^-permeable channels [11]. In the skeletal muscles of *mdx* mice, a model for Duchenne muscular dystrophy (DMD), the protein expression of Ca^2+^-permeable receptor-activated channel, TRPC3, is increased [12,13,14]. In mice overexpressing skeletal muscle-specific TRPC3, Ca^2+^ influx and skeletal muscle atrophy were exacerbated [14,15]. In addition, excess ROS production, driven by upregulation of Nox2, is also associated with muscle atrophy [16,17,18].

In the heart composed of striated muscles, physiological stretching activates Nox2 on cardiomyocyte membranes, inducing ROS production [19]. Increased ROS activates ryanodine receptor 2 (RyR2), leading to a local increase in intracellular Ca^2+^ concentration through oxidative modification of RyR2 (X-ROS signaling) [19,20,21]. We have previously reported that TRPC3 amplifies X-ROS signaling through formation of a protein complex between TRPC3 and Nox2 in pressure overload-induced myocardial remodeling and doxorubicin-induced myocardial hypertrophy in mice [22,23]. By forming the TRPC3-Nox2 complex through the TRPC3 C-terminal region, Nox2 is stably retained at the cell membrane and escapes from proteasome-dependent degradation, which leads to increased intracellular ROS level [22]. Furthermore, we found that pyrazole-3 (Pyr3), a TRPC3-selective blocker, and ibudilast, an anti-asthma drug, inhibit the formation of the TRPC3-Nox2 complex [24,25].

While the relationship between increased intracellular Ca^2+^ influx and ROS generation in muscle atrophy has been discussed, the involvement of the TRPC3-Nox2 complex in skeletal muscle atrophy remains unclear. Compared with the heart, which is composed of a homogenous group of striated myocardial cells, skeletal muscles consist of a mixture of various phenotypes of striated muscle cells, such as red muscle, white muscle, and stem cells. In this study, we aim to investigate the formation of the TRPC3-Nox2 complex and its involvement in the pathogenesis of skeletal muscle atrophy.

## 2. Methods and Materials

### 2.1. Materials

Ibudilast was purchased from Tokyo Chemical Industry Co., Ltd. (Tokyo, Japan). Pyr3 was purchased from Selleck Co. (Kanagawa, Japan).

### 2.2. Data Validation

The Gene Expression Omnibus (GEO) database was accessed. The gene expression profiling of muscle samples from patients with Myotonic dystrophy type 1 (DM1) (GSE85984) and Collagen VI-related muscular dystrophies (COL6RDs) (GSE103608) [26], a rare hereditary congenital muscular dystrophy, was reanalyzed to compare TRPC3 and Nox2 expression levels. Similarly, *mdx* mouse data (GSE162455, GSE178772) were also reanalyzed [27,28]. From data of GSE162455, we used data from the soleus muscle of 5-month-old male *mdx* mice (C57BL/10ScSn-*Dmd^mdx^*/J) and their background C57BL/10ScSn/J mice. In GSE178772, the tibialis anterior muscle profiling of 6-month-old male wild-type (WT) (C57/BL6) and *mdx*^5cv^ mice, which were also kept on the C57BL/6J strain, was reanalyzed.

### 2.3. Animals

All protocols using mice were approved by the Animal Care and Use Committee, Kyushu University, or followed the guidelines of National Institutes of Natural Sciences and were performed according to Institutional Guidelines Concerning the Care and Handling of Experimental Animals (protocol code: A20-150-0 approved on 13 January 2020, A21-154-0 approved on 27 November 2020, A23-164-1 approved on 29 October 2024, A24-423-0 approved on 11 August 2024). Animal studies are reported in compliance with the ARRIVE guidelines [29]. Laboratory animals were randomly assigned to experimental groups, and treatments were assessed blindly. The order of treatment administration was also randomized. All animal samples were studied, and analysis was carried out in a blinded manner.

The 129Sv background mice and the TRPC3 (−/−) mice were maintained and bred in Kyushu University. The 4-week-old male, C57BL/10-*mdx* mice (11–18 g, *n* = 39 in total) were purchased from CLEA, Inc. (Tokyo, Japan). The 4-week-old male, C57BL/10 mice (12–19 g, *n* = 29 in total) were purchased from SLC (Shizuoka, Japan). An osmotic pump that slowly releases ibudilast (10 mg/kg/day), Pyr3 (0.1 mg/kg/day), or vehicle (50% (*v*/*v*) DMSO/50% (*v*/*v*) PEG300) was implanted in the abdominal cavity of the 4-week-old male C57BL/10 mice or C57BL/10-*mdx* mice. Ibudilast and Pyr3 were also dissolved in the solvent (50% (*v*/*v*) DMSO/50% (*v*/*v*) PEG300). All mice were sampled after muscle strength measurement.

All mice were maintained in specific-pathogen-free areas (light/dark cycle 12 h/12 h, room temperature 21–23 °C, and humidity 50–60%) and group-housed (*n* = 2–4 per cage) in a clear plastic cage (15 × 30 × 15 cm) and given free access to food and water. The tibialis anterior muscle (TA), extensor digitorum longus (EDL), gastrocnemius muscle (GM), SM, quadriceps muscle (QM), and heart were isolated in each mouse. Plasma creatine kinase (CK) was measured with dry clinical chemistry analysis (dry-Chem, Fujifilm, Tokyo, Japan).

### 2.4. Denervation Animal Model

The 8-week-old male, 129Sv mice (21–24 g, *n* = 12), and TRPC3 (−/−) mice (22–24 g, *n* = 12) were anesthetized with isoflurane. The sciatic nerve of the right hind leg was exposed, and about 5 mm of its length was cut off, and then the skin was sutured. All mice were sampled 2 weeks after surgery.

### 2.5. Muscle Strength Measurement

The hanging wire test was conducted to measure muscle strength pre- and post-surgery. Each mouse was placed on a wire mesh elevated 40 cm high and inverted upside down [30]. The time it took the mouse to fall was recorded. The test was repeated at three-minute intervals for a total of five trials, and the average of the five measurements was taken as the measurement value.

The grip test was conducted to measure muscle strength. Grip strength was measured using a grip strength measuring device (GPM-101B, MELQUEST, Toyama, Japan) pre- and post-surgery. The mouse was held by its tail and allowed to grasp the horizontal wire mesh of this device. Once a firm grip was established, the mouse was lightly pulled backward by its tail until it released the wire mesh. The maximum grip strength (Max power) was measured when the mice released the wire mesh.

In denervation model mice, muscle strength was measured 2 weeks after surgery. In *mdx* mice, muscle strength was measured 4 weeks after administration.

### 2.6. Immunohistochemistry

During sampling, heart and skeletal muscle tissues were embedded in O.C.T. compound (Cat # 90501, Sakura Finetek, Torrance, CA, USA) and frozen in isopentane mixed with dry ice. Thin sections (12 μm) were prepared from frozen heart and skeletal muscle using a Cryostat (Thermo Fisher Scientific, Waltham, MA, USA), dried for 1 h, washed twice with PBS, stained with 5 μM dihydroethidium (DHE), and incubated for 30 min at 37 °C, and then washed with PBS 3 times. Sections were then fixed with 4% paraformaldehyde for 10 min at room temperature and washed twice with PBS. Tissues were stained with Alexa Fluor 488 conjugated Wheat Germ Agglutinin (WGA) (Invitrogen, Eugene, OR, USA, 1:500) and 4′,6-diamidino-2-phenylindole dihydrochloride (DAPI) (Dojindo, Kumamoto, Japan, 1:1000) for 1 h at room temperature under light-shielded conditions. The slides were mounted with cover glass after washing twice with PBS and immediately analyzed through BZ-X800 microscopy (Keyence, Osaka, Japan) and an LSM900 (ZEISS, Oberkochen, Germany). ImageJ software (1.54p) was used to quantify the cellular cross-sectional area (CSA) and fluorescence intensity of DHE in myocytes. At least five sections were imaged for each muscle tissue. Since each image contained over 20 muscle fibers, we measured at least 100 fibers.

Frozen mouse skeletal muscle sections (12 μm thickness) were dried for 1 h and then fixed with 4%PFA for 10 min. Immunoblocking was performed with PBS containing 0.1% Triton X-100 and 1% BSA for 1 h at room temperature. Primary antibodies against Nox2 (Proteintech, Rosemont, IL, USA, RRID: AB_2833044, 1:400) and CD45 (BioLegend, San Diego, CA, USA, RRID: AB_312966, 1:100) were incubated with the tissue overnight at 4 °C, and then the sections were washed with PBS 3 times. Tissues were then incubated with Alexa 647 conjugated WGA (Invitrogen, Eugene, OR, USA, 1:500) and fluorescence-labeled secondary antibody (Alexa488 goat anti-rat IgG, Thermo Fisher Scientific, Waltham, MA, USA, Cat#A11006, RRID: AB_2534074, and Alexa594 goat anti-rabbit IgG: Thermo Fisher Scientific, Waltham, MA, USA, Cat#A11037, RRID: AB_2534095, 1:1000) and DAPI for 1 h at room temperature under light-shielded conditions. Slides were then washed twice with PBS and mounted with a cover glass. Imaging was observed on an LSM900 (ZEISS, Oberkochen, Germany). At least five fields of view were randomly taken and analyzed for each mouse.

### 2.7. Proximity Ligation Assay (PLA) to Detect TRPC3-Nox2 Interaction

The TRPC3 and Nox2 interaction in mouse skeletal muscle was visualized using a Duolink PLA kit (Sigma Aldrich, Burlington, MA, USA) according to the manufacturer’s instruction. Frozen skeletal muscles were cut using a Cryostat (Thermo Fisher Scientific, Waltham, MA, USA) to a thickness of 14 μm, which was optimal for this assay. After fixing and blocking, muscles were incubated with mouse anti-Nox2 antibody (Proteintech, Rosemont, IL, USA, RRID: AB_2833044, 1:10) and rabbit anti-TRPC3 antibody (Santa Cruz Biotech, Dallas, TX, USA, sc-514670, 1:150) for 48 h at room temperature, followed by PLA probe incubation for 1 h. The ligation (1 h) and amplification (3 h) steps were performed in a 37 °C chamber, and these tissues were stained with DAPI and WGA. Images were observed on an LSM900 (ZEISS, Oberkochen, Germany). At least five fields per mouse were randomly taken, and PLA signals were counted from these images.

### 2.8. Real-Time RT-PCR

Total RNA was isolated from frozen skeletal muscles using the RNeasy Fibrous Tissue Mini Kit (QIAGEN, Hilden, Germany) according to the manufacturer’s instructions. Complementary DNA (cDNA) was synthesized with Prime Script RT (Takara Bio, Kusatsu, Shiga, Japan). Real-time PCR was performed using the ΔΔCt method. The 18S rRNA was used as the internal control. The primers are described in Supplementary Table S1.

### 2.9. Expression of TRPC3 C-Terminal Peptide by AAV

A peptide fragment (C-terminal peptide: 53 amino acids) that interacts with Nox2 at the C-terminal region of TRPC3 was amplified using PCR and incorporated into the pEGFP-N1 vector [22]. The C-terminal peptide (TRPC3-C-GFP) fused with EGFP under the control of the CAG promoter was subcloned into pZac2.1 (Penn Vector). AAV 2/9 vectors containing CAG promoter encoding AcGFP or TRPC3-C-GFP were constructed according to a previously reported method [31]. AAV (9 × 10^10^ genomic copies) was administered by injection into the gastrocnemius muscle of 4-week-old male WT and *mdx* mice. Muscle strength was measured 4 weeks after administration, and skeletal muscles were then harvested to confirm the expression of AcGFP and TRPC3-C-GFP using an LSM900 (ZEISS, Oberkochen, Germany).

### 2.10. Statistical Analysis

All results are presented as the mean ± SEM and were considered significant if *p* < 0.05. Data normality was assessed using the Shapiro–Wilk test. For datasets that did not significantly deviate from normality, parametric tests were applied. Comparisons of means between two groups were performed by Student’s *t*-test and the Mann–Whitney test for normally and not normally distributed samples, respectively. For three or more groups, a two-way analysis of variance (ANOVA) followed by Tukey’s post hoc test was performed for normally distributed samples. Multiple comparison testing for non-normally distributed data was performed using the nonparametric Kruskal–Wallis test. Statistical analysis was performed using GraphPad Prism 8.0 (GraphPad Software, LaJolla, CA, USA).

## 3. Results

### 3.1. Nox2 Expression Increases in Atrophic Skeletal Muscle

**Figure 1:**
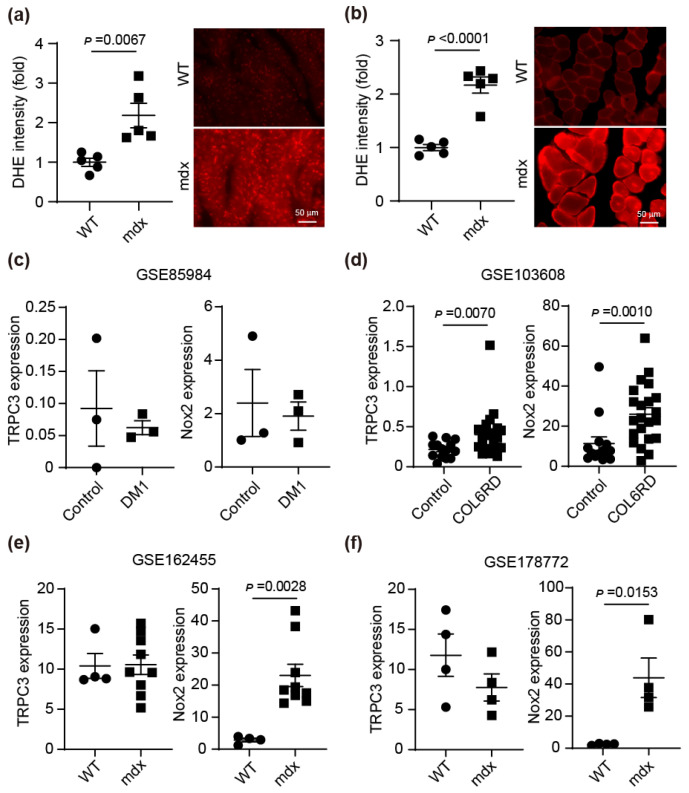
Nox2 expression level increases in atrophic skeletal muscle. (**a**,**b**) Counting result (left) and representative images (right) of ROS production in heart (**a**) and SM (**b**) of *mdx* mice. Scale bar = 50 μm, *n* = 5 mice/each group. (**c**–**f**) RNA-seq profiles of GREIN in healthy control and muscle atrophy patients or mice. Comparison of TRPC3 and Nox2 mRNA expression levels in skeletal muscle of control patients and myotonic dystrophic (DM1) patients (**c**), control patients and patients with collagen VI-related muscular dystrophies (COL6RDs) (**d**), and WT and *mdx* mice (**e**,**f**). The numbers at the top of the graphs indicate the accession numbers. All data are shown as mean ± SEM; significance was analyzed using a *t*-test (**a**–**c**,**f**) or Mann–Whitney test (**d**,**e**) for true two-group comparison.

The involvement of TRPC3 and Nox2 in muscle atrophy has been reported fragmentarily in mice and rats [12,15]. In DMD, cardiac weakness and skeletal muscle atrophy have been reported [30,31]. In the heart and skeletal muscle of *mdx* mice, a mouse model of DMD, ROS production was increased (Figure 1a,b). Next, we reanalyzed data from the database to determine whether the expression of these genes changes in muscle atrophy in humans and mice. Although the number of subjects was only three, there were no changes in the expression levels of TRPC3 and Nox2 in skeletal muscle between control patients and myotonic dystrophic (DM1) patients (Figure 1c). Skeletal muscle in patients with collagen VI-related muscular dystrophies (COL6RDs) showed elevated TRPC3 and Nox2 mRNA levels (Figure 1d). Similarly, comparison of skeletal muscle between control mice and *mdx* mice revealed an increased expression level of Nox2 (Figure 1e,f). These results suggested that Nox2 expression increases with skeletal muscle atrophy, consistent with previous reports [17,18].

### 3.2. TRPC3 Deletion Attenuates Denervation-Induced Soleus Atrophy

**Figure 2:**
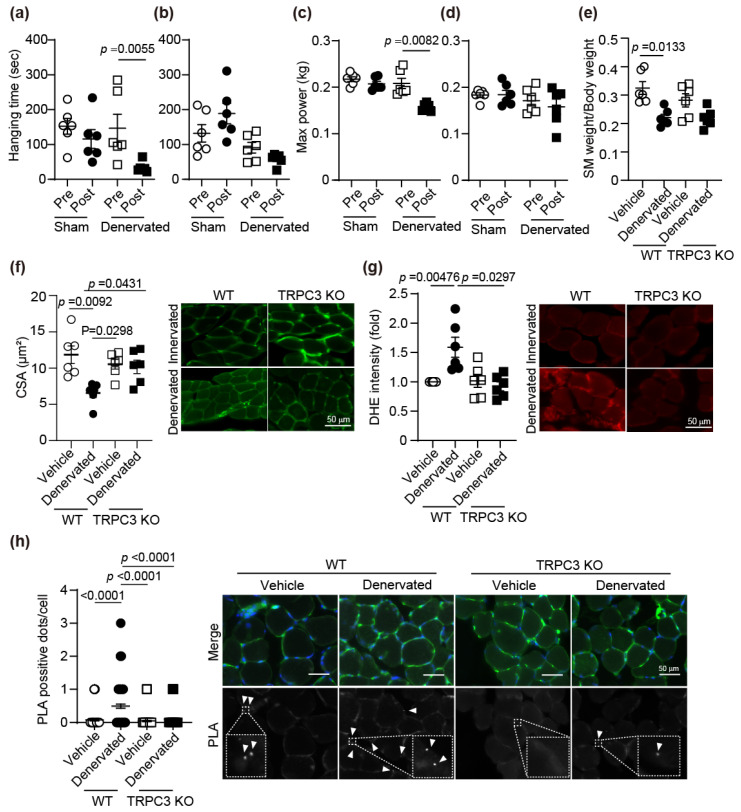
Deletion of TRPC3 attenuates sciatic denervation-induced muscle atrophy. (**a**–**d**) Muscle strength of sham and denervated WT (**a**,**c**) and TRPC3 KO mice (**b**,**d**) 2 weeks after surgery. Hanging time (**a**,**b**) and Max power (**c**,**d**) were measured at the point of pre-surgery (Pre) and post-surgery (Post). (**e**,**f**) The weight (**e**) and CSA (**f**) of SM in WT and TRPC3KO mice treated with and without denervation. Cell surface was stained with WGA. (**g**) Representative images and counting result of denervation-induced ROS production in SM. (**h**) PLA signals between TRPC3 and Nox2 are shown as white spots (arrowheads), counterstained with WGA (green) and DAPI (blue). Average number of PLA signals for each cell was quantified in left panel. The area enclosed by the white dashed line has been enlarged. At least 50 cells were counted. Scale bar = 50 μm. All data are shown as mean ± SEM; *n* = 6 mice/each group. Data were analyzed using two-way ANOVA followed by Tukey’s comparison test (**a**–**g**) or Kruskal–Wallis test (**h**) for multi-group panels.

Muscular atrophy caused by nerve transection is a major contributor to the decline in physical function observed with aging [32,33]. As a muscle atrophy model mouse mimicking age-related muscle atrophy, we used sciatic nerve transection mice. This model allows us to verify skeletal muscle atrophy innervated by the transected sciatic nerve. In the hanging wire test and grip test, which measure muscle strength, the time that WT mice could cling to the wire was shortened, and muscle strength decreased after surgery, but no significant difference was observed in TRPC3-deficient (TRPC3 KO) mice (Figure 2a–d). The weight of TA, EDL, GM, and SM in WT mice decreased due to denervation (Figure 2e and Figure S1a). In TRPC3 KO mice, the weight of other muscles reduced compared with that before treatment, but the weight of SM did not change (Figure 2e and Figure S1a). There was no significant change in the weight of QM in both groups (Figure S1a). As with the weight of SM, the CSA of SM in denervated-WT mice decreased, but there was no change in TRPC3 KO mice (Figure 2f). The CSA of TA, EDL, and GM decreased in both WT and TRPC3 KO mice following denervation, and CSA of QM remained unchanged in both groups regardless of denervation (Figure S1b). In WT mice, the amount of ROS in SM increased after denervation, but ROS did not change in TRPC3 KO mice (Figure 2g). ROS production also increased in other skeletal muscles of denervated mice (Figure S1c). Furthermore, the PLA-positive signals indicating the TRPC3-Nox2 protein complex were significantly increased in the SM of denervated-WT mice compared with those in control mice, and these signals did not increase in TRPC3 KO mice regardless of denervation treatment (Figure 2h). These results reveal that TRPC3-Nox2 complex formation is involved in muscle atrophy, especially in the SM.

### 3.3. Ibudilast Inhibits TRPC3-NOX2 Complex Formation and Ameliorates Muscle Wasting

**Figure 3:**
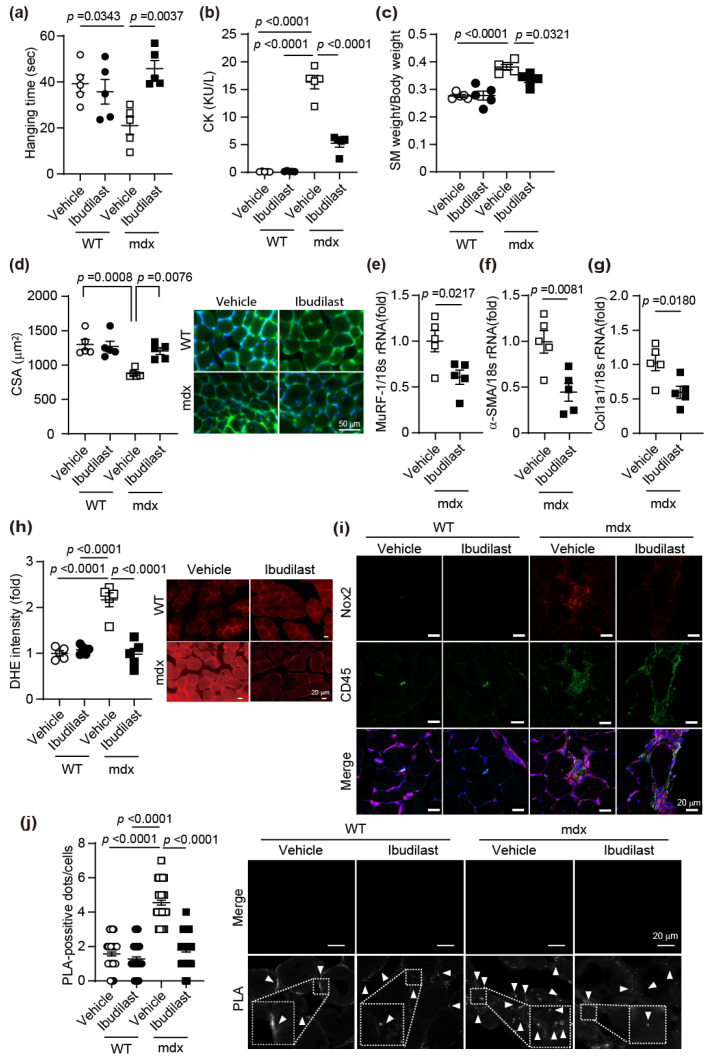
Ibudilast suppresses muscle atrophy in *mdx* mice by inhibiting TRPC3-Nox2 complex formation. (**a**) Muscle strength of vehicle or ibudilast-treated WT and *mdx* mice. (**b**) The effect of ibudilast on plasma CK in WT and *mdx* mice. (**c**) Muscle weight in SM of WT and *mdx* mice treated with ibudilast. (**d**) CSA of SM in vehicle or ibudilast-treated WT and *mdx* mice. Cell morphology was stained with WGA and DAPI. Scale bar = 50 μm. (**e**–**g**) mRNA expression levels of MuRF-1 (**e**), α-SMA (**f**), and Col1a1 (**g**) in the SM of *mdx* mice. (**h**) Counting result (left) and representative images (right) of ROS production of SM in WT and *mdx* mice. (**i**) The localization of Nox2 (red), CD45 (green), DAPI (blue), and WGA (purple) in SM. (**j**) PLA signals between TRPC3 and Nox2 are shown as white spots (arrowheads). Average number of PLA signals for each cell was quantified in left panel. The area enclosed by the white dashed line has been enlarged. At least 50 cells were counted. Scale bar = 20 μm. All data are shown as mean ± SEM; *n* = 5 mice/each group. Significance was analyzed using two-way ANOVA followed by Tukey’s comparison test (**a**–**d**) or Kruskal–Wallis test (**j**) for multi-group panels, and *t* test for true two-group comparisons (**e**–**g**).

It has been demonstrated that the formation of the TRPC3-Nox2 complex is involved in muscle atrophy induced by denervation. Next, we evaluated whether the formation of the TRPC3-Nox2 complex is involved in muscle atrophy in *mdx* mice, using ibudilast, a TRPC3-Nox2 complex formation inhibitor. Ibudilast had no effect on the hanging time of WT mice but significantly increased the hanging time of *mdx* mice (Figure 3a). CK levels in the blood of *mdx* mice, a marker of muscle damage, were elevated compared with WT mice, whereas ibudilast significantly decreased CK levels in the ibudilast-treated group compared with those in the vehicle-treated group of *mdx* mice (Figure 3b). The body weight and the muscle weights of TA, EDL, GM, and QM of *mdx* mice treated with vehicle were not significantly different from those in the ibudilast-treated group (Figure S2). However, ibudilast significantly suppressed the increase in muscle weight of SM in *mdx* mice (Figure 3c). CSA of vehicle-treated *mdx* mice significantly reduced, but ibudilast suppressed this reduction (Figure 3d). Ibudilast suppressed the mRNA expression level of MuRF1, which is one of the muscle atrophy markers, in *mdx* mice (Figure 3e). The expression level of α-SMA and COL1A1, the fibrosis markers, was reduced in ibudilast-treated *mdx* mice (Figure 3f,g). ROS production, which increased in the SM of *mdx* mice, was suppressed by ibudilast (Figure 3h). Furthermore, in SM of *mdx* mice, CD45-positive hematopoietic cells migrate into the intercellular spaces, and Nox2 expression increases, but ibudilast suppressed this (Figure 3i). The number of PLA-positive signals in *mdx* mice was significantly increased in the SM compared with those in WT mice. Ibudilast suppressed this increase (Figure 3j). These results indicate that TRPC3-Nox2 complex formation is involved in muscle atrophy.

### 3.4. TRPC Inhibitor Pyr3 Reproduces These Protective Effects

**Figure 4:**
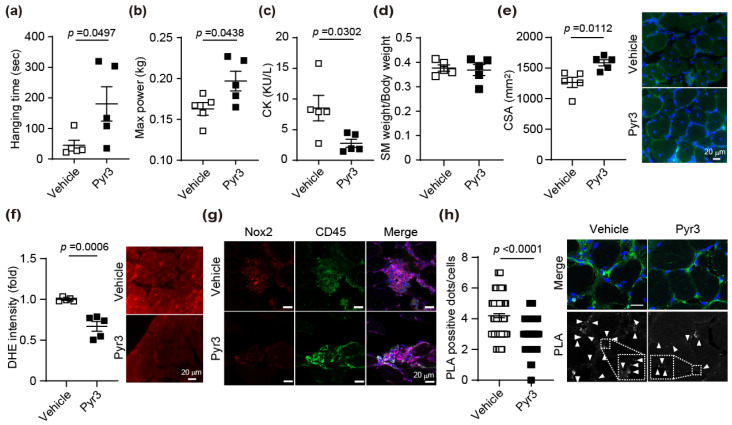
Pyr3 suppresses muscle atrophy due to the inhibition of TRPC3-NOX2 complex formation in *mdx* mice. (**a**,**b**) Muscle strength in vehicle or Pyr3-treated WT and *mdx* mice. (**a**) Hanging time and (**b**) Max power. (**c**) The effect of Pyr3 on plasma CK in WT and *mdx* mice. (**d**) Muscle weight in SM of WT and *mdx* mice treated with Pyr3. (**e**) SM of Pyr3-treated *mdx* mice was stained with WGA and DAPI, and the CSA was measured. (**f**) Counting result (left) and representative images (right) of ROS production of SM in Pyr3-treated *mdx* mice. (**g**) The localization of Nox2 (red), CD45 (green), DAPI (blue), and WGA (purple) in SM. (**h**) PLA signals between TRPC3 and Nox2 are shown as white spots (arrowheads), co-stained with WGA (green) and DAPI (blue). Average number of PLA signals for each cell was quantified in left panel. The area enclosed by the white dashed line has been enlarged. At least 50 cells were counted. Scale bars = 20 μm. All data are shown as mean ± SEM; *n* = 5 mice/each group. Significance was analyzed using a *t*-test (**a**–**f**) or Mann–Whitney test (**h**) for true two-group comparisons.

Ibudilast not only inhibits TRPC3-Nox2 complex formation but also inhibits phosphodiesterase 4 (PDE4) [34]. Therefore, we used Pyr3, a TRPC3 inhibitor that more specifically inhibits this complex formation, to evaluate whether the TRPC3-Nox2 complex is involved in muscle atrophy [25]. Measurements were taken 4 weeks after the drug administration. Hanging time and maximal grip strength (Max power) were significantly increased in Pyr3-treated *mdx* mice (Figure 4a,b). CK levels were significantly decreased in Pyr3-treated group than in the vehicle group (Figure 4c). The body weight and muscle weights of TA, EDL, GM, SM, and QM of *mdx* mice treated with vehicle were not significantly different from those in Pyr3-treated group (Figure 4d and Figure S3a–e). *Mdx* mice treated with Pyr3 had significantly increased CSA compared with the vehicle group (Figure 4e). ROS production in SM was suppressed by Pyr3-treatment (Figure 4f). Pyr3 suppressed the expression of Nox2 in CD45-positive blood cells infiltrating skeletal muscle cells in *mdx* mice (Figure 4g). The SM of *mdx* mice treated with Pyr3 significantly reduced the number of PLA-positive signals compared with the vehicle group (Figure 4h). These results suggest that inhibition of complex formation between TRPC3 and Nox2 by Pyr3 prevents muscle atrophy.

### 3.5. AAV-Mediated TRPC3 C-Terminal Peptide Expression Suppresses Muscle Atrophy Locally

**Figure 5:**
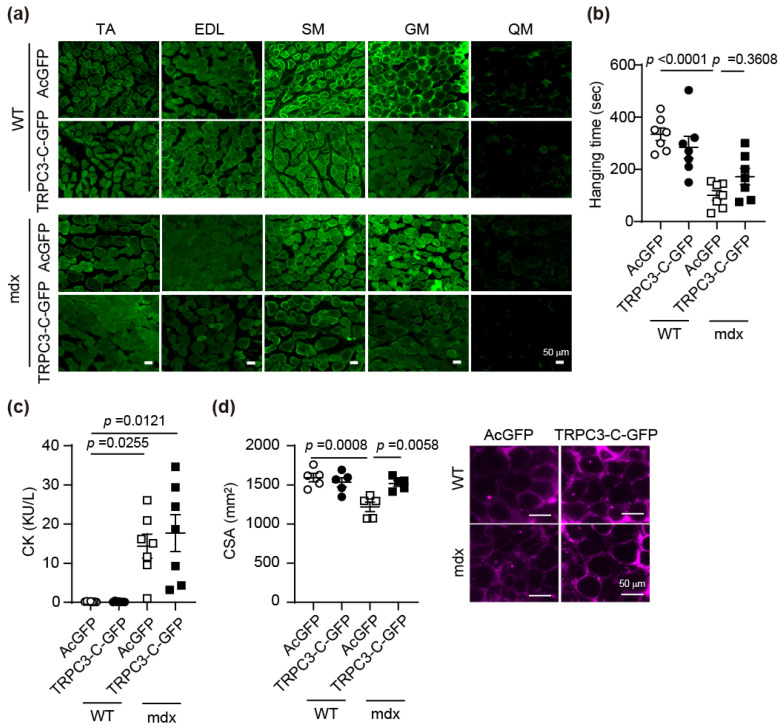
Expression of TRPC3 C-terminal peptides in skeletal muscle suppresses muscle atrophy in mdx mice. (**a**) The expression level of GFP in each AVV-injected skeletal muscle. Scale bar = 50 μm. (**b**) Hanging time in AAV-injected mice. (**c**) The effect of TRPC3 C-terminal peptide on plasma CK in WT and *mdx* mice. (**d**) The CSA of SM in TRPC3 C-terminal peptide expressed in mice compared with WT mice. Scale bar = 50 μm. All data are shown as mean ± SEM; *n* = 7/each group. Significance was analyzed using two-way ANOVA followed by Tukey’s comparison test (**b**,**d**) or the Kruskal–Wallis test (**c**).

Pharmacological inhibition of TRPC3-Nox2 complex formation improved muscle atrophy. Next, to verify the efficacy of enhancing muscle atrophy from an endogenous source, the C-terminal peptide of TRPC3, which inhibits TRPC3-Nox2 complex formation, was overexpressed in the hindlimb skeletal muscle of WT and *mdx* mice by AAV. This C-terminal protein does not affect Ca^2+^ influx through TRPC3 even when overexpressed and has been shown to inhibit ROS production mediated via Nox2 [22]. WT and *mdx* mice administered each AAV showed no change in body weight. Although the muscles of *mdx* mice were significantly larger, there was no difference in muscle weight between AcGFP and TRPC3 C-terminal peptide (Figure S4). This peptide was expressed in the TA, EDL, and SM, including the injection site GM, but not in the QM (Figure 5a). In *mdx* mice expressing TRPC3-C-GFP, hanging time was not increased (Figure 5b). CK showed no significant difference between AcGFP and TRPC3-C-GFP groups of *mdx* mice (Figure 5c). CSA was reduced compared with that of WT mice in *mdx* mice and was restored in *mdx* mice expressing TRPC3-C-GFP (Figure 5d). These results suggested that inhibition of the TRPC3/Nox2 interaction by the C-terminal peptide suppressed muscle atrophy.

## 4. Discussion

Muscular dystrophy causes not only skeletal muscle atrophy but also cardiac dysfunction [35]. Indeed, detection of ROS levels within muscle tissue of *mdx* mice revealed elevated levels in both cardiac and skeletal muscle (Figure 1). Reanalysis of several databases showed an upward trend in Nox2 expression in atrophied skeletal muscle from both mice and humans (Figure 1). In denervated mouse models, muscle atrophy occurred in the muscles innervated by the sciatic nerve, but no muscle atrophy was observed in the QM, which is not innervated by the sciatic nerve. However, ROS production increased similarly to that in other muscles (Figure S1). In the SM of TRPC3 nerve-resected mice, neither muscle atrophy nor increased ROS production was observed (Figure 2). In WT SM from nerve-transected mice, the formation of the TRPC3-Nox2 complex was promoted, but the formation of the TRPC3-Nox2 complex was not detected in TRPC3 KO mice.

Compared with control mice, *mdx* mice showed a significant increase in muscle weight in the TA and SM (Figure 3 and Figure S2). The increase in weight is thought to be due to the infiltration of blood cells into the gaps between atrophied muscle cells [36]. Treatment with ibudilast suppressed the increase in SM weight. On the other hand, Pyr3 did not result in any difference in the weight of SM in *mdx* mice (Figure 4d). *Mdx* mice have been shown to exhibit anti-fibrotic and anti-inflammatory effects through the inhibition of PDE4 and PDE5, leading to prevention of muscle atrophy [37,38]. The effects of ibudilast, which is also a PDE4 inhibitor, are not only due to the inhibition of TRPC3-Nox2 complex formation but also due to systemic PDE inhibition. These results suggest that the anti-inflammatory effects of ibudilast suppressed blood cell infiltration and reduced SM weight. However, since there was no significant difference in phenotypic improvement effects between ibudilast and Pyr3 treatment, these results suggest that the TRP3-Nox2 complex plays a major role in DMD.

The results of this study suggest that the contribution of TRPC3-Nox2 complex formation to muscle atrophy is particularly high in SM among the five muscles. Skeletal muscles are known to exhibit heterogeneity in terms of muscle fiber types and metabolic characteristics. SM is mainly composed of type 1 fibers, which belong to red muscle. Therefore, it functions as a slow-twitch muscle and has been reported to have oxidative metabolism [3,39]. Other muscles mainly use glycolytic metabolism and are either white muscle or a mixture of white and red muscle. Cardiac muscle is the same type of red muscle as SM, and we previously reported that hypoxia-inducible factor (HIF)-1α expression increased in doxorubicin-induced shrunk heart tissue, along with an increase in Nox2 expression levels [23]. HIF-1α is known to enhance ROS production by increasing expression of Nox2 [40,41]. Since SM is oxygen-dependent, it is in a hypoxic state during muscle atrophy [42], and it is considered that Nox2 contributes more than in other muscles. Therefore, it is suggested that inhibiting TRPC3-Nox2 complex formation was more effective in SM atrophy. It has been reported that inducing pharmacologically atrophied muscles to differentiate slow-twitch and oxidative muscle fibers and activating them is beneficial for improving skeletal muscle function [43]. This supports the results of this study regarding SM.

In skeletal muscle, Nox2 is located along the sarcolemma and in the t-tubules [17,44]. The phosphorylation of p47*^phox^*, an essential scaffold protein in Nox2, occurs in the muscles of *mdx* mice and is an important step in the activation of Nox2 [18]. Furthermore, Ang2-induced skeletal muscle atrophy was not induced in Nox2 KO mice [45]. These reports support the notion that ROS production mediated by Nox2 could be a promising therapeutic target. In skeletal muscle fibers of mice overexpressing TRPC3 specifically in skeletal muscle, calcium entry was dramatically increased, and skeletal muscle atrophy was induced [15]. It has been reported that the protein expression level of TRPC3 is significantly increased in the skeletal muscle of DMD rats [12]. When AAV-microdystrophin was overexpressed throughout the body of DMD rats to suppress muscle dysfunction, TRPC3 protein expression levels decreased, suggesting that TRPC3 may be involved in intracellular calcium overload associated with DMD pathogenesis [12]. Nox2 and TRPC3, which have been reported to be involved in muscle atrophy, have been found in this study to interact with each other and cause skeletal muscle atrophy. Furthermore, infiltration of CD45-positive blood cells was observed in the SM of *mdx* mice. Although the expression level of Nox2 was increased in these cells, ibudilast and Pyr3 tended to suppress Nox2 expression (Figure 3i and Figure 4g). Since TRPC3 is widely expressed in blood cells, it is considered that the formation of the TRPC3-Nox2 complex was also promoted in these blood cells, similar to the cell membranes of skeletal muscle [46,47]. During muscle injury, macrophages and neutrophils initially migrate to the site [48]. Subsequently, satellite cell activation promotes muscle fiber regeneration [36]. Since ROS production plays crucial roles in inflammation, cell migration, and adhesion [49], the increased Nox2 expression in CD45-positive hematopoietic cells is thought to be important for the initial response to muscle injury. While it is known that Nox2 is expressed in leukocytes and neutrophils within CD45-positive hematopoietic cells, the specific type of blood cell involved in muscle atrophy remains unclear [50,51]. We plan to determine which blood cell type exhibits the most significant TRPC3-Nox2 complex formation contributing to muscle atrophy. Furthermore, to clearly distinguish the physiological role of TRPC3/Nox2 in skeletal muscle from its role in CD45-positive blood cells, site-specific knockout analysis of either of these genes will be necessary in future studies.

Recently, gene therapy has been used to treat muscle dystrophy [4]. We overexpressed TRPC3 C-terminal peptide (53 amino acids), which inhibits TRPC3-Nox2 complex formation, in the hindlimbs using AAV, and evaluated the effects on the phenotype in *mdx* mice. GFP was expressed in TA, EDL, SM, and GM, and since AAV was administered via intramuscular injection into the hindlimbs, it is suggested that AAV reached these muscles. It is presumed that AAV did not reach the QM, which is distant. In *mdx* mice expressing this peptide, there was no improvement in muscle strength or CK (Figure 5b,c), but muscle atrophy was suppressed (Figure 5d). These results suggest that the expression of this peptide was limited to part of the hindlimbs and did not inhibit the systemic formation of the TRPC3-Nox2 complex, unlike when ibudilast or Pyr3 was administered.

It was previously known that mechanical stretch stimuli induce ROS production from Nox2, which activates RyR2 and increases local intracellular Ca^2+^ concentration through the X-ROS signaling in the heart [19,20]. We previously reported that the formation of the TRPC3-Nox2 complex is promoted in pathological conditions, leading to increased ROS production and causing myocardial atrophy and reduced flexibility [23,25]. X-ROS signaling is thought to be deeply involved in this mechanism. Therefore, the formation of the TRPC3-Nox2 complex on the cell membrane under pathological conditions is thought to be the cause of the inability of stretch-sensitive cells, such as cardiomyocytes and skeletal muscle cells, to adapt flexibly to stretching. In the future, it will be necessary to elucidate the mechanism by which this interaction is formed. Currently, the treatment for muscular dystrophy includes exon skipping therapy and symptomatic treatment with steroids. Exon skipping therapy is limited to specific patients [4]. While DMD is the type used in this study, various other types of muscular dystrophy exist. Although the causative genes vary widely, the processes following muscle degeneration and necrosis share high commonality, resulting in similar clinical symptoms. Therefore, inhibiting TRPC3-Nox2 complex formation is considered applicable to a broad range of muscle atrophy, including sarcopenia, and could represent a novel therapeutic strategy.

## 5. Conclusions

**Figure 6:**
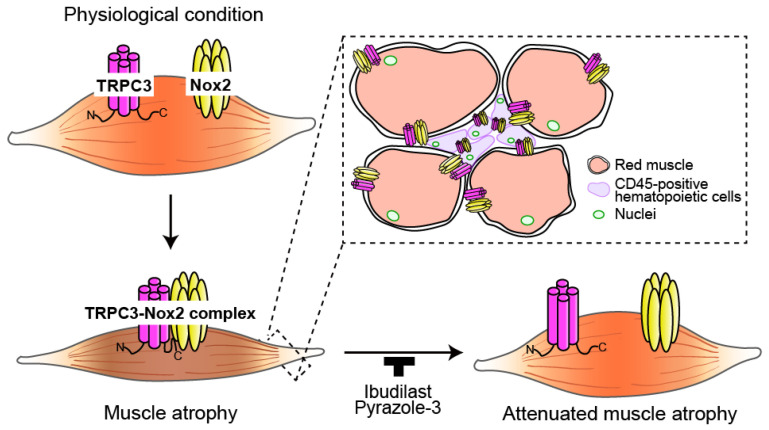
TRPC3-Nox2 complex formation contributes to the progression of skeletal muscle atrophy. The interaction between TRPC3 and Nox2 is enhanced in SM and CD45-positive hematopoietic cells under pathological conditions. This leads to increased ROS production, which causes muscle atrophy. Muscle atrophy is suppressed by the pharmacological inhibition of TRPC3-Nox2 complex formation by ibudilast and Pyr3. N indicates the N-terminal and C indicates the C-terminal of TRPC3.

Although TRPC3 and Nox2 had been reported to participate in skeletal muscle atrophy, their interactions have not been discussed. We revealed that the formation of the TRPC3-Nox2 complex contributes to the progression of muscle atrophy in denervated mice and DMD model mice and that pharmacological inhibition of TRPC3-Nox2 complex formation prevents muscle weakness (Figure 6).

TRPC3-Nox2 complex formation is a pivotal driver of ROS-mediated skeletal muscle atrophy. Targeting this protein–protein interaction represents a promising therapeutic strategy for Duchenne muscular dystrophy and other intractable muscle-wasting disorders.

## Data Availability

The original contributions presented in this study are included in the article. Further inquiries can be directed to the corresponding author.

## Supplementary Materials

The following supporting information can be downloaded at https://www.mdpi.com/article/10.3390/antiox15010038/s1.

## Abbreviations

The following abbreviations are used in this manuscript:

AAV	adeno-associated virus
CSA	cross-sectional area
CK	creatine kinase
DHE	dihydroethidium
DAPI	4′,6-diamidino-2-phenylindole dihydrochloride
DMD	Duchenne muscular dystrophy
EDL	extensor digitorum longus
GM	gastrocnemius muscle
HIF	hypoxia-inducible factor
mROS	mitochondrial ROS
Nox	NADPH oxidase
PDE	phosphodiesterase
Pyr	pyrazole
QM	quadriceps muscle
ROS	reactive oxygen species
TRPC	transient receptor potential canonical
KO	knock out
TA	tibialis anterior muscle
RyR	ryanodine receptor
WGA	wheat germ agglutinin
WT	wild type
SM	soleus muscle
