# Supplementary material for: Inhibition of TRPC3-Nox2 Complex Formation Ameliorates Skeletal Muscle Atrophy
Source: Antioxidants (Basel). 2025 Dec 26;15(1):38. doi: 10.3390/antiox15010038 (PMC12838269; doi:10.3390/antiox15010038)
Supplement: Supplementary file 1 [file antioxidants-15-00038-s001.zip › antioxidants-4019478-supplementary.pdf]

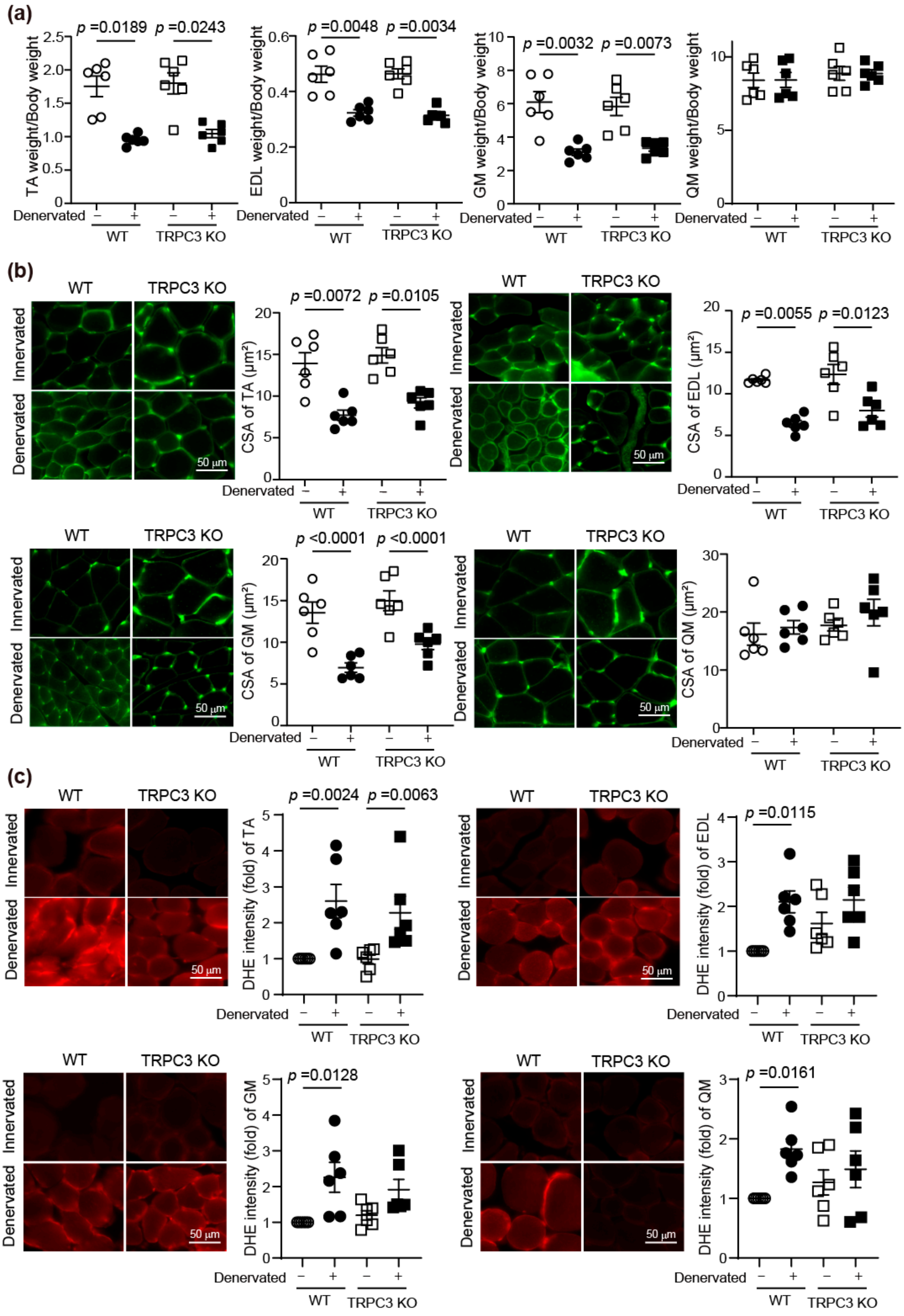
(a)
p =0.0189 p =0.0243
TA weight/Body weight
Denervated
WT
TRPC3 KO
p =0.0048 p =0.0034
EDL weight/Body weight
p =0.0032 p =0.0073
GM weight/Body weight
QM weight/Body weight
(b)
WT TRPC3 KO
Innervated
Denervated
50 μm
p =0.0072 p =0.0105
CSA of TA (μm²)
p =0.0055 p =0.0123
CSA of EDL (μm²)
p <0.0001 p <0.0001
CSA of GM (μm²)
CSA of QM (μm²)
(c)
p =0.0024 p =0.0063
DHE intensity (fold) of TA
p =0.0115
DHE intensity (fold) of EDL
p =0.0128
DHE intensity (fold) of GM
p =0.0161
DHE intensity (fold) of QM

Supplementary Figure S1. The effects on other skeletal muscles in denervated mice

(a) Muscle weight, (b) CSA, and (c) ROS production in TA, EDL, GM, and QM of WT and TRPC3 KO mice in Figure 2. All data are shown as mean ± SEM; n=6. Data were analyzed using two-way ANOVA followed by Tukey's comparison test.

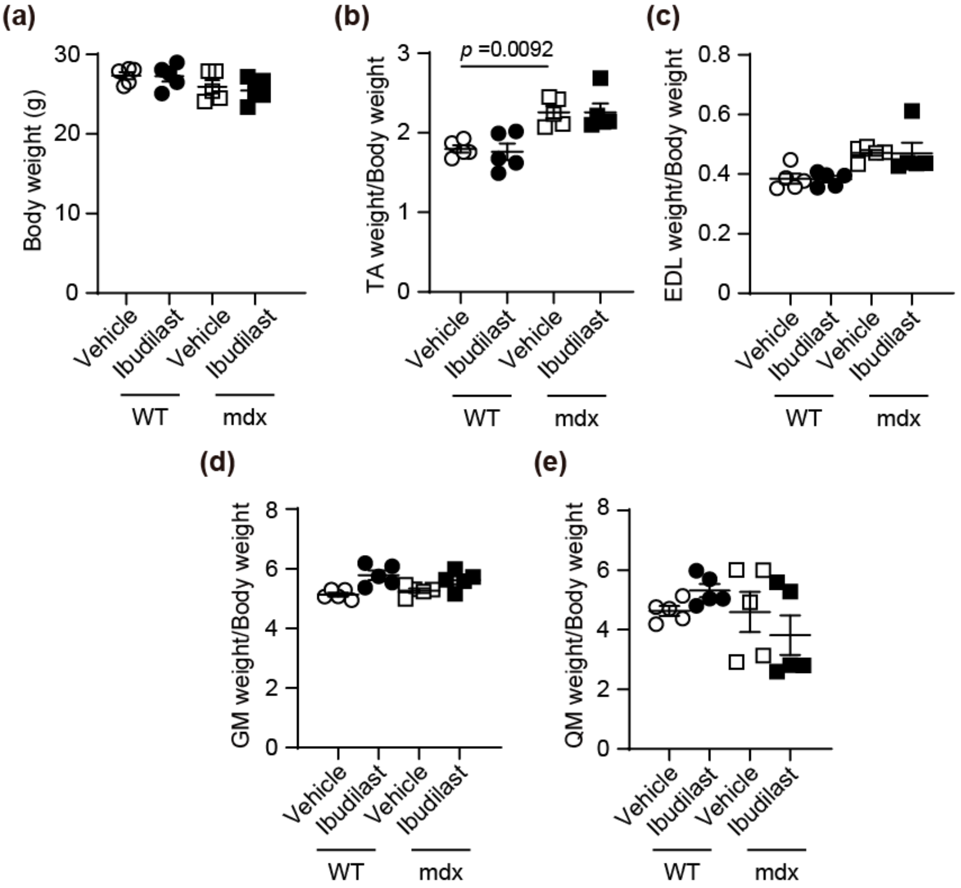

Supplementary Figure S2. Comparison of skeletal muscle weight in mdx mice treated with vehicle or Ibudilast.

(a) Body weight of all mice. (b-f) Muscle weight in TA(b), EDL(c), SM(d), GM(e), and QM(f) of WT and mdx mice treated with Ibudilast in Figure 3. All data are shown as mean ± SEM; n=5 mice. Significance was analyzed using two-way ANOVA followed by Tukey's comparison test.

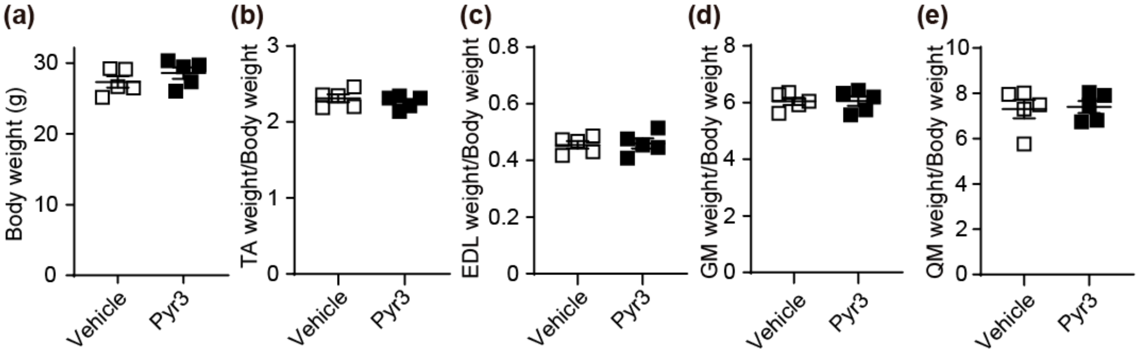

Supplementary Figure S3. Comparison of skeletal muscle weight in mdx mice treated with vehicle or Pyr3.

(a) Body weight of all mice. (b-f) Muscle weight in TA(b), EDL(c), GM(d), and QM(e) of mdx mice treated with Pyr3 in Figure 4. All data are shown as mean ± SEM; n=5 mice. Significance was analyzed using t-test.

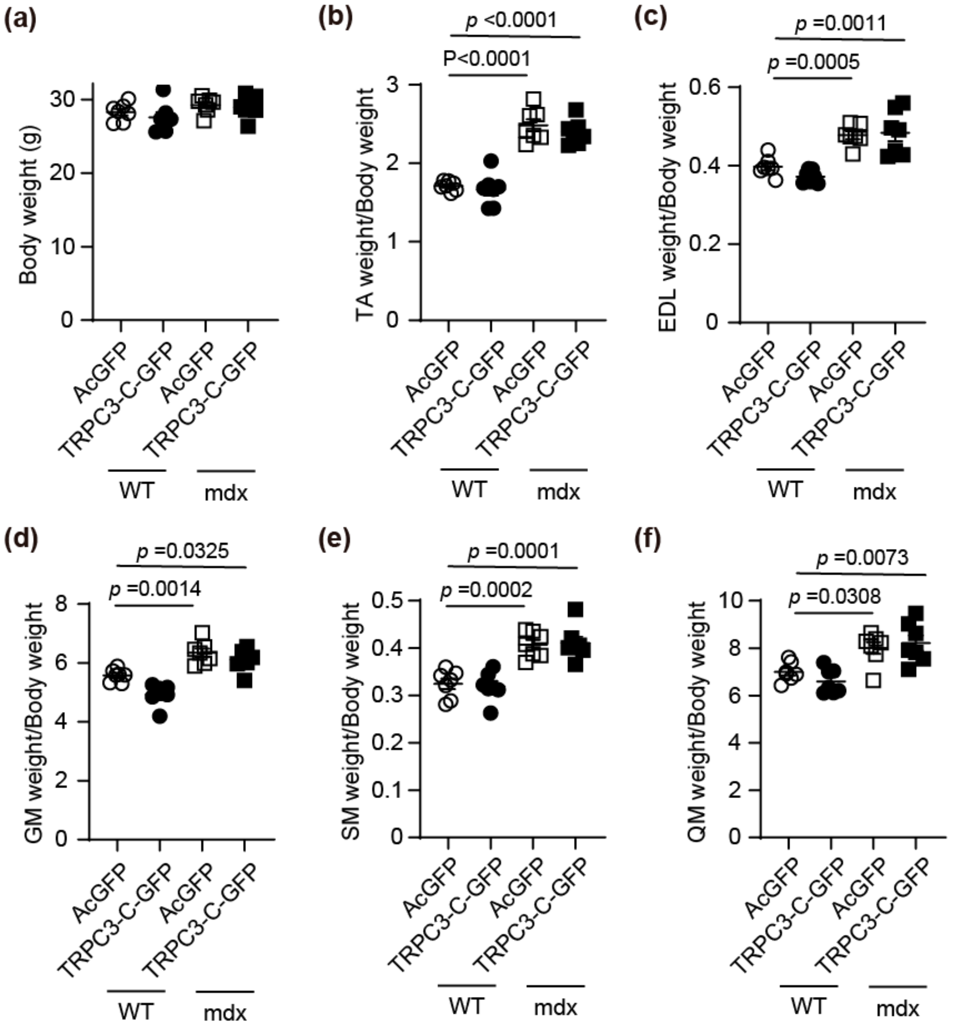

Supplementary Figure S4. Comparison of skeletal muscle weight in AAV-injected WT and mdx mice

(a) Body weight of all mice. (b-f) Muscle weight in TA (b), EDL (c), SM (d), GM (e), and QM (f) of mdx mice in Figure 5. All data are shown as mean ± SEM; n=7 mice. Significance was analyzed using two-way ANOVA followed by Tukey's comparison test.

Supplementary Table S1. Primer List

No.	Gene		Primer Sequence (5' - 3')
1	Mouse α-SMA	Forward	GTCCCAGACATCAGGGAGTAA
		Reverse	TCGGATACTTCAGCGTCAGGA
2	Mouse MuRF-1	Forward	GTGTGAGGTGCCTACTTGCTC
		Reverse	GCTCAGTCTTCTGTCCTTGGA
3	Mouse Col1a1	Forward	GCTCCTCTTAGGGGCCACT
		Reverse	CCACGTCTCACCATTGGGG
4	18s rRNA	Forward	ATTAATCAAGAACGAAAGTCGCAGGT
		Reverse	TTTAAGTTTCAGCTTTGCAACCATACT
